# Evaluation of endophytic colonization and establishment of entomopathogenic fungi against *Tuta absoluta *(Lepidoptera: Gelechiidae) in tomato plants

**DOI:** 10.12688/f1000research.148658.1

**Published:** 2024-07-16

**Authors:** Dereje Geremew, Tadale Shiberu, Ararsa Leta

**Affiliations:** 1 Ethiopian Institute of Agricultural Research, Ambo Agricultural Research Center, Ambo, Ethiopia; 2 Ambo University, Guder Mamo Mezemir Campus, College of Agriculture, Ambo, Ethiopia; 3 Ambo University, Guder Mamo Mezemir Campus, College of Agriculture, Ambo, Ethiopia

**Keywords:** Beauveria bassiana, Colonization, Endophytic, entomopathogenic fungi, Metarhizium robertsii, Tuta absoluta

## Abstract

**Background:**

The tomato, Solanum lycopersicum L., is one of the most important horticultural crops that can be consumed fresh or after being processed worldwide. The tomato leaf miner (Tuta absoluta) is one of the most devastating pest to tomato plants due to its mine-feeding nature in the mesophyll tissue of the plant. Fungal entomopathogens can exist naturally in plants as an asymptote. This study aimed to detect the endophytic colonization of Beauveria bassiana and Metarhizium robertsii within tomato plants via artificial inoculation and their virulence effects on Tuta absoluta.

**Methods:**

Isolates with the highest percent germination and virulence against T. absoluta were selected for endophytic evaluation within tomato plants by different artificial inoculation techniques.

**Results:**

This study revealed that, isolates with the highest percent germination and virulent to Tuta absoluta had the potential to colonize tomato plants. The result showed that, the maximum mortality rate (97.5%) of Tuta absoluta larvae was achieved by Metarhizium robertsii isolate K-61 at a concentration of 1x10 ^8^conidial/ml at 7 days post inoculated. However, the highest cumulative mortality (100%) was recorded by Beauveria bassiana isolate APPRC-27 at 10 days post inoculated through the direct contact method. The highest endophytic colonization was registered by isolate APPRC-27 (76.67%) at 7 days post-inoculated using the leaf spray technique, but it declined to 11.67% after 28 days of inoculated. In the case of the seedling inoculation technique, the highest endophytic colonization was obtained in the root tissues of tomatoes at 28 days of inoculated by isolate K-61.

**Conclusions:**

This study revealed that the leaf spray inoculation technique was the most effective method, followed by seedling inoculation, for the deployment of Beauveria bassiana and Metarhizium robertsii endophytes in tomato plant tissues. Therefore, virulent Beauveria bassiana and Metarhizium robertsii, are promising bioagents for the control of Tuta absoluta if deployed as endophytes.

## Introduction

The tomato, *Solanum lycopersicum* L., is one of the most cultivated vegetables worldwide due to its low-fat content and excellent source of dietary fiber, minerals, vitamins, and antioxidants ( Erdinc *et al.,* 2018). It can be consumed fresh and/or processed into a wide variety of manufactured products ( Sousa *et al*., 2008). World-wide tomato cultivation suffers enormous losses due to insect attacks, which reduce the quality of the fruits and spread plant pathogens. The South American tomato pinworm, *Tuta absoluta* (Meyrick) (Lepidoptera: Gelechiidae), is native to South America and one of the primary global pests of tomatoes that causes 80–100% yield losses ( Desneux *et al*., 2010). The pest first arrived in Ethiopia in 2012 via Sudan and later expanded to other parts of the country ( Mansour *et al.*, 2018; Shiberu & Getu, 2017; Gashawbeza and Abiy, 2013).

In Ethiopia, the yield loss due to *T. absoluta* was reported in the range of 60.08% to 82.31% ( Shiberu & Getu, 2018). It also reduces the quality of the yield, which directly affects marketability, and causes secondary infection by creating wounds. This increases the cost of production to farmers, which they pay for pesticides in the country, particularly in West Shewa. Considering alternative ways of protecting EPF from adverse conditions, the incorporation of EPF in plants as endophytes would appear to be a highly interesting and promising approach ( Vega, 2018).

The endophytes contribute to plant protection by controlling herbivores, insects, and pathogens on the host plant. In addition, they may strengthen plant resistance to pathogens and biotic and abiotic stresses ( Ahlholm *et al*., 2002; Kogel *et al*., 2006). As a result of direct contact with potential insect hosts, entomopathogenic fungi (EPF) infect their hosts by penetrating through cuticular exoskeletons ( Zimmermann, 2007).

Endophytic microorganisms are now attracting great interest from researchers as an alternative source for the control of plant pests. The introduction of entomopathogenic fungi as endophytes could protect the pathogen from adverse environmental conditions, reducing the negative effects of ultraviolet radiation and the limitations of low humidity on development. Entomopathogenic fungi could be deployed as a biological control strategy against specific pests, especially those with life cycles that require feeding inside leaves, stems, rhizomes, roots, and seeds, behaviors that reduce their exposure to synthetic insecticides and other control methods ( Resquín-Romero *et al*., 2016).

Currently, EPF is more promising than chemical pesticides for the control of pests in Ethiopia due to pesticide resistance. Colonization of a host plant by an endophyte is influenced by the inoculation method, species of fungal endophytes, and the host plant species itself. Based on the inoculation technique, the endophytes differ in their ability to colonize different plant parts and persist over a crop growth cycle ( Akello *et al*., 2007; Brownbridge *et al.,* 2012). Knowing the endophytic association and establishment of EPF in tomato plants helps to modify the inoculation techniques for plant parts based on the behavior of targeted pests, particularly *T. absoluta*, which lives in the mesophyll tissue of the tomato plant. However, such successful work is not available in Ethiopia on endophytic colonization of EPF on tomato plants against *T. absoluta.* Therefore, this study was conducted to detect endophytic colonization of *B. bassiana* and *M. robertsii* with tomato plants by different inoculation techniques and their virulence effect against *T. absoluta.*

## Methods

### Description of the study area

The experiment was conducted at the Ambo Agriculture Research Center under laboratory and glasshouse conditions. Ambo is far away from Addis Ababa, 110 km to the west, at a geographical coordinate of 8°59’ N latitude and 37.85°E longitude, with an altitude of 2100 meters above sea level. Daily temperature ranges between 15°C-29°C and annual 22°C on average.

### Conidial suspension preparation

Conidia were obtained from pure cultures grown on the 65g of Sabouraud Dextrose Agar (SDA) Microxpress ^®^ REF: 201190030500 which were incubated for 14 days at 27±1°C under dark conditions. Conidia were harvested by disposable cell scrapers and added into test tubes containing tween 80 (0.001%v/v). Suspensions were vortexed well for 2 min and adjusted to 1 × 10 ^8^ conidia/ml using a Neubauer haemocyutometer ( Gurulingappa *et al*., 2010; Hassan *et al*., 2019; Ozdemir *et al*., 2021).

### Insect rearing

The mass rearing of *T. absoluta* was established in 2021 from larvae collected from tomato plants without any history of pesticides in fields surrounding Ambo areas. The Roman VF variety tomato plant was grown in the glasshouse to maintain the life cycle of *T. absoluta.* The host plant, the tomato, was maintained under quarantine to discard the presence of potential diseases and parasites. Groups of four cages (height: 30 cm; diameter: 23 cm) were used for rearing, each with one tomato plant. The first cage was used for adult oviposition, the second for 1 ^st^ to 2 ^nd^ instar larvae, the third for 3 ^rd^ to 4 ^th^ instars, and the fourth cage for maintaining the pupae and emerging adults. Larvae were fed pesticide-free tomato seedlings ( *S. Lycopersicum cv.* Roman VF), whereas adults were fed a 15% honey-water solution ( Allegrucci *et al.,* 2017). The maintenance of the insects and mass rearing were conducted under glasshouse conditions at Ambo Agricultural Research Center (AmARC).

### Screening the virulence of isolates against *T. absoluta*

High-virulent *B. bassiana* and *M. robertsii* isolates that were prescreened on *G. melonella* were again tested against 2 ^nd^ larval instars of *T. absoluta* ( Sabbour and Singer, 2014). Five *B. bassiana* and three *M. robertsii* isolates were tested at a concentration of 1 × 10 ^8^ conidial/ml for the screening of virulence against 2 ^nd^ instar larvae of *T. absoluta.* Briefly, spore suspensions were adjusted to 1 × 10 ^8^ conidia/ml using Tween 80 (0.001%v/v) in sterile distilled water.

Tomato leaves Surface disinfection was done by washing with 1% sodium hypochlorite for 2 min, 2 min in ethanol (70%), and rinsing 3 times with sterile distilled water. Surface-sterilized leaves were placed on sterile plastic plates, sprayed with 3 ml of fungal spores, and air-dried under the safety cabinet for 3 minutes. Ten second-instar larvae of *T. absoluta were* separately released over leaves and incubated for 7 days at room temperature. A control treatment was sprayed with Tween 80 (0.001%) and incubated under the same conditions.

The mortality of larvae was recorded daily, and dead larvae were surface sterilized and placed on sterile plastic plates containing moistened filter paper. Plates with larval cadavers were moistened daily with distilled sterile water to enhance mycosis development over the cadavers and incubated at room temperature.

Mycosis percentages were calculated using the percentage of cadavers showing external fungal growth out of the total number of tested insects ( Sabbour, 2014). The experiment was conducted under laboratory conditions in the CRD with nine treatments and replicated four times.

### Evaluation of the virulence of fungal isolates against *T. absoluta*

To determine the infectivity of the fungal isolates against *T. absoluta*, four isolates were evaluated by the direct contact method ( Allegrucci *et al.,* 2017). Three *M. robertsii* isolates, namely K-63, K-61, and K-102, with one *B. bassiana APPRC-27, were* selected based on their conidial viability, yield, production, and radial growth for their virulence against *T. absoluta,* according to Dotaona *et al.* (2015), Campos-Herrera *et al*. (2021), and Gebremariam *et al*. (2021).

Surface disinfected 400 tomato leaf discs of approximately 2 cm diameter were cut, and 320 of them were immersed 0.001% in solution of Tween 80 of *B. bassiana* and *M. robertsii* (1 × 10 ^8^ conidia/ml) separately, while the other 80 (controls) were immersed in a 0.001% conidia-free solution of Tween 80. The experiment was conducted in the laboratory by CRD with five treatments and replicated four times, with each treatment containing 20 2 ^nd^ instar larvae. Leaf discs were placed on a plastic plate separately, with a second instar larva per disc. Every 24 hours, larval mortality was recorded. Larval mortality was evaluated daily for 10 consecutive days. Dead larvae were removed and the surface disinfected.

Data on mortality were corrected for their control mortality by using Abbot’s ( Abbott, 1925) formula:

$$\text{Percent of}\;\text{Corrected mortality}=\frac{\left(\%T-\%C\right)}{\left(100-\%C\right)}\times 100$$

Where: CM=Corrected mortality, C, mortality in the untreated larvae, and T is mortality in the treated larvae.

The cadavers were placed in sterile Petri dishes with filter paper moistened with sterile distilled water, and mycosis was confirmed under a stereomicroscope by microscopical examination of the dead insects ( Allegrucci *et al.,* 2017).

### Preparation of the host plant tomato for the assessment of colonization endophytic fungi

***Tomato seed surface sterilization***

The Roman VF variety was brought from Melkassa ARC and sown in the glasshouse at AmARC. Before sowing, the seeds were surface sterilized by successive immersion in 1% sodium hypochlorite for 2 minutes, then 70% alcohol for 3 minutes, and finally three washes in sterile distilled water ( Brownbridge *et al*., 2012). Seeds were then placed on sterile filter paper to dry for 30 minutes and divided into two portions. One portion was used for seedling inoculation techniques, and the other was used for leaf spraying and root dipping after seedling emergence.

***Growing the tomato plants***

Seeds were planted for each inoculation technique separately, and pots were filled with compost, loam soil, and sandy soil in the ratio of 1:1:2, respectively.

The soil was sterilized in an autoclave for 6 hours at 121°C before use at Ambo University’s Mamo Mezmir campus. The experiment was conducted under glasshouse conditions at 25–30°C, and all necessary agronomic practices were followed for the growth of tomato plants. Treatments were arranged in CRD and replicated four times for each inoculation technique. A total of 576 tomato seeds were sown, and all agronomic practices were done as recommended.

### Evaluation of inoculation techniques

The most virulent isolates of *B. bassiana* and *M. robertsii* against *T. absoluta* were selected for the examination of endophytic colonization in tomato plants. Two *M. robertsii* and one *B. bassiana* isolate were tested with three inoculation techniques (leaf spray, root dipping, and seedling inoculation) for the presence or absence of endophytic colonization of these fungi in tomato plant tissues. Endophytic colonization and establishment potential of *B. bassiana* and *M. robertsii* isolates were evaluated by introducing them into tomato plants through seedling inoculation, root dipping, and leaf spraying techniques ( Allegrucci *et al*., 2017; Russo *et al*., 2019; Silva *et al*., 2020). A total of 480 tomato plants were randomly chosen and used for the detection of endophytic colonization.

***Leaf spraying***

The leaves of tomato plants (7 weeks old) were sprayed with three ml of 1 × 10 ^8^ conidia ml ^–1^ suspension in a glass hand sprayer (30 ml capacity). To avoid conidial runoff into the soil, the top of each pot was covered with aluminum foil ( Posada *et al*., 2007). Control plants were sprayed with a conidia-free solution of 0.001% Tween 80 ( Saragih, 2019; Sinno *et al*., 2021).

***Seedling inoculation***

Endophytic colonization of tomato seedlings with isolates was conducted by inoculating seedlings with *B. bassiana* and *M. robertsii* conidia. The seeds were surface disinfected in 1% sodium hypochlorite for 2 minutes, followed by 70% ethanol for 2 minutes, and rinsed 3 times with sterile distilled water ( Arnold and Herre, 2003; Posada and Vega, 2006). Surface-sterilized seeds were placed in plastic Petri dishes lined with filter paper, moistened with 1 ml sterilized distilled water, and placed at 70% relative humidity for 6 days to stimulate germination. *B. bassiana* and *M. robertsii* Conidial suspension was added to germinated seeds at 1 × 10 ^8^ conidia/ml with tween 80 (0.001%v/v). Two days post-inoculation of fungus, the seedlings were transferred to pots filled with sterilized soil and planted carefully without damaging the roots. For control treatment, surface sterilization was conducted as mentioned above, and control seeds were treated with tween 80 (0.001%v/v) 6 days post-germination ( Silva *et al*., 2020).

***Root dipping***

For root dipping, three weeks of seedlings were used after emergence. Each seedling was removed from the pot and rinsed three times with sterile distilled water. The ends of the roots were cut for better absorption and placed individually in test tubes with 2 ml of a conidial suspension containing 1 × 10 ^8^ conidia/ml ( Akello *et al*., 2007; Bamisile et *al.*, 2018; Allegrucci *et al*., 2020). Each tube was covered with aluminum foil to prevent damage from UV rays. The roots of control plants were submerged in sterile distilled water. Control and treated plants were incubated at 26°C with 70% relative humidity and under a 12:12 (L:D) photoperiod for 24 h. Thereafter, both the control and treated plants were placed on sterile filter paper until completely dried and then replanted in the same pots. Seedlings in all treatments were watered as needed and maintained in a glasshouse.

### Evaluation of endophytic colonization of fungi

For each inoculation technique, Colonization of tomato plants by *B. bassiana* and *M. robertsii* was assessed at 7, 14, 21, and 28 DPI. The evaluation was conducted for the presence or absence of *B. bassiana* and *M. robertsii* conidia on 480 plants in total, 120 plants for each inoculation technique, and 40 plants for each isolate (360 treated plants and 120 control plants). Ten plants were randomly chosen at each re-isolation time and taken to a laboratory for the detection of endophytic colonization of isolates through plant tissues. Ten replications were made for each plant part (leaf, stem, and root), and 30 replications were used for each isolate.

Accordingly, tomato plants were surface disinfected as stated above, and complete disinfection of leaves was checked by plating 100 ml of the last rinsing water of each sample onto SDA ( Gurulingappa *et al*., 2010). Plant parts were dried on sterile filter paper under a laminar flow cabinet, and their edges were cut to remove dead tissue due to the sterilization process.

Examination of conidia was conducted by cutting plant parts (leaf, stem, and root) into six small pieces, approximately 5 mm ^2^ for leaf and 5mm for stem and root, by using surgical blades; 2880 pieces were used in total, then placed on a Petri dish containing SDA with antibiotic ( Vega *et al*., 2008a).

Confirmation was conducted by visual observation under a stereomicroscope for the presence or absence of fungi after 10 days of incubation of the culture at 27 °C. For each inoculation technique, the colonization percentage was calculated as follows ( Greenfield *et al*., 2016).

$$\%\text{Colonization}=\frac{\text{Number of pieces exhibiting fungal growth}\;}{\text{Total number of pieces}\;}\times 100$$

### Data analysis

Data on larval mortality was calculated using Abbot’s formula (1925). Means were separated using Tukey’s Honestly Significant Difference (HSD) at P 0.05 for screening against *T. absoluta* and evaluation of isolates. The mean colonization (expressed in percentage) data were arcsine transformed to stabilize the efficacy of the analysis of variance ( Gomez and Gomez, 1984).

## Results and Discussions

### Results

***Screening the virulence of isolates against T. absoluta larvae***

The result revealed that there is a highly significant difference among isolates in the mean percent mortality of *T. absoluta* larvae (F = 67.89; DF = 8; P < 0.0001). Isolate K-61 showed the highest mortality rate (97.5%) against the larval stage of *T. absoluta* under laboratory conditions after 7 days of inoculation ( Table 1). The lowest mortality rate was registered by APPRC-44BC and K-5 isolates (72.5%).

**Table 1.** A mortality rate of larvae and their mycosis percentage.

Isolates	Mortality ±SE at 7 DPI	Mycosis %±SE
*B. bassiana APPRC-*44BC	72.5±2.50 ^c^	25.00±2.89 ^cd^
*M. robertsii K*-63	95±2.89 ^ab^	60.00±0.00 ^ab^
*B. bassiana K*-5	72.5±4.79 ^c^	20.00±0.00 ^d^
*M. robertsii* K-102	92.5±2.5 ^ab^	55.00±2.85 ^b^
*B. bassiana APPRC*-27	90.00±4.08 ^ab^	62.5±2.5 ^ab^
*B. bassiana K*-91	85.00±2.89 ^abc^	65.00±2.89 ^ab^
*B. bassiana RST*-8	80.00±4.08 ^bc^	32.5±2.50 ^c^
*M. robertsii* K-61	97.5±2.50 ^a^	67.5±2.50 ^a^
Control (tween 80)	7.5±2.89 ^d^	0.00±0.00 ^e^
HSD at (0.05)	16.11	10.14
CV (%)	8.71	9.81

**Note:** Means with the same letter in the same column are not significantly different according to Tukey’s Studentized Range (HSD) test at, α = 0.05.

**Figure 1.**
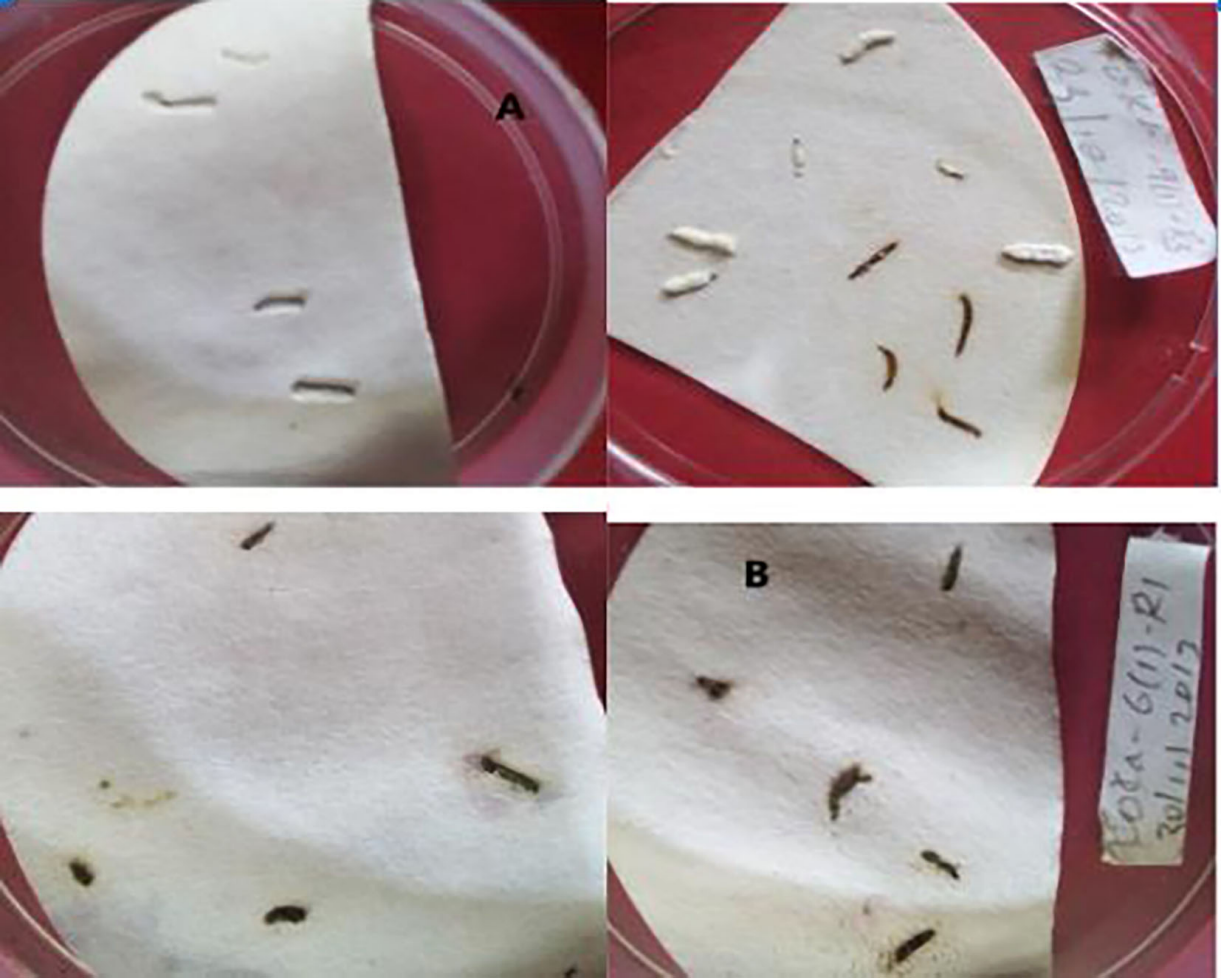
Mycosis of *B. bassiana* and *M. robertsii* isolates over cadavers. A = *B. bassiana*, B = *M. robertsii.*

Fungal mycosis was developed on the dead cadavers of *T. absoluta* larvae, which were able to select the most virulent isolates against *T. absoluta* ( Figure 1). There is a highly significant difference among treatments in mycosis percentage on dead cadavers, and no mycosis was observed in the control treatment ( Table 1).

A higher mycosis percentage was obtained from K-61 and K-91, with 67.5% and 65%, respectively. The minimum mycosis percentage was recorded by K-5 (20%), followed by APPRC-44BC with 25% on cadavers of *T. absoluta* larvae.

### Evaluation of selected isolates against *T. absoluta* by direct contact method

**Figure 2.**
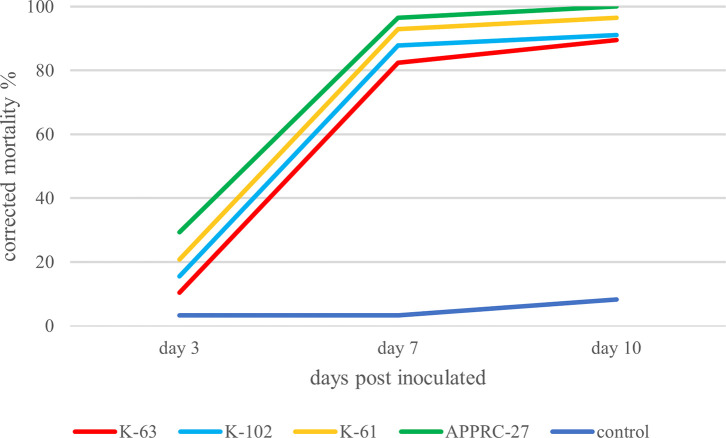
Cumulative mortality of larvae.

This result showed that all isolates were highly virulent to the larval stage of *T. absoluta* under laboratory conditions at 1 × 10 ^8^ conidial/ml after 10 days of inoculation. The highest cumulative mortality (100%) was obtained from *B. bassiana* isolate APPRC-27 ( Figure 2). According to the present study, *B. bassiana* and *M. robertsii* were able to infect the larval stage of *T. absoluta* by direct contact method.

***Median lethal time (LT _50_)***

This study revealed that fungal isolates with the highest mortality rate against *T. absoluta* record the lowest LT _50_ days, and vice versa. As a result, the lowest median lethal time (LT _50_) and (LT _90_) of *T. absoluta* larvae were attained by *B. bassiana* isolate *APPRC*-27 with 3.67 and 6.19 days, respectively, at 1 × 10 ^8^ conidia/ml ( Table 2).

**Table 2.** Median lethal time (LT50) and (LT90) of *T. absoluta* larvae treated with isolates 10 days post inoculated at 1 × 10 ^8^ conidia/ml.

Fungal isolates	LT _50_(days) (95% CL)±SE	LT _90_(days) (95% CL)±SE	Chi-square	Intercept
*M.* robertsii K-63	4.96±0.10	9.29±0.28	2.20	-3.30
*M.* robertsii K-102	4.62±0.15	8.32±0.29	2.23	-1.16
*M.* robertsii K-61	3.94±0.04	7.2±0.4	2.52	-2.29
*B. bassiana APPRC*-27	3.67±0.04	6.19±0.22	3.32	3.24

### Endophytic colonization of fungal isolates in tomato plants

***Leaf spray***

The result showed that a higher percentage of colonization was obtained at 7 DPI by *B. bassiana* isolate APPRC-27 with 76.67%, 35.00%, and 16.67% in leaves, stems, and roots, respectively, through the leaf spray method of inoculation ( Table 3)

**Table 3.** Endophytic colonization of isolates in tomato plants by leaf spray technique.

Isolate	Days	Leaf spray		
		Leaves	Stems	Roots
*M.* robertsii *K*102	7	50.00 ^b^	16.26 ^b^	8.33 ^b^
	14	26.67 ^c^	10.00 ^c^	8.33 ^b^
	21	15.33 ^b^	7.16 ^b^	8.33 ^a^
	28	3.33 ^c^	3.33b	0.00b
*M.* robertsii *K*61	7	53.33 ^b^	21.67 ^b^	10.00 ^b^
	14	35.00 ^b^	18.33 ^a^	13.33 ^a^
	21	29.67 ^a^	11.16 ^a^	5.50 ^b^
	28	10.00 ^b^	8.33 ^a^	3.34 ^a^
*B. bassiana APPRC27*	7	76.67 ^a^	35.00 ^a^	16.67 ^a^
	14	54.00 ^a^	15.00 ^b^	5.00 ^c^
	21	28.33 ^a^	6.86 ^b^	1.67 ^c^
	28	11.67 ^a^	1.67 ^c^	0.00 ^b^
*Control*	7	0.00 ^c^	0.00 ^d^	0.00 ^c^
	14	0.00 ^d^	0.00 ^d^	0.00 ^d^
	21	0.00 ^c^	0.00 ^c^	0.00 ^d^
	28	0.00 ^d^	0.00 ^d^	0.00 ^b^

.

The lowest endophytic colonization was recorded after 28-day inoculation with 11.67% (SE±3.56) in leaves and 1.67% (SE±1.67) stems; no *B. bassiana* was detected in tomato roots at 28 DPI.

**Figure 3.**
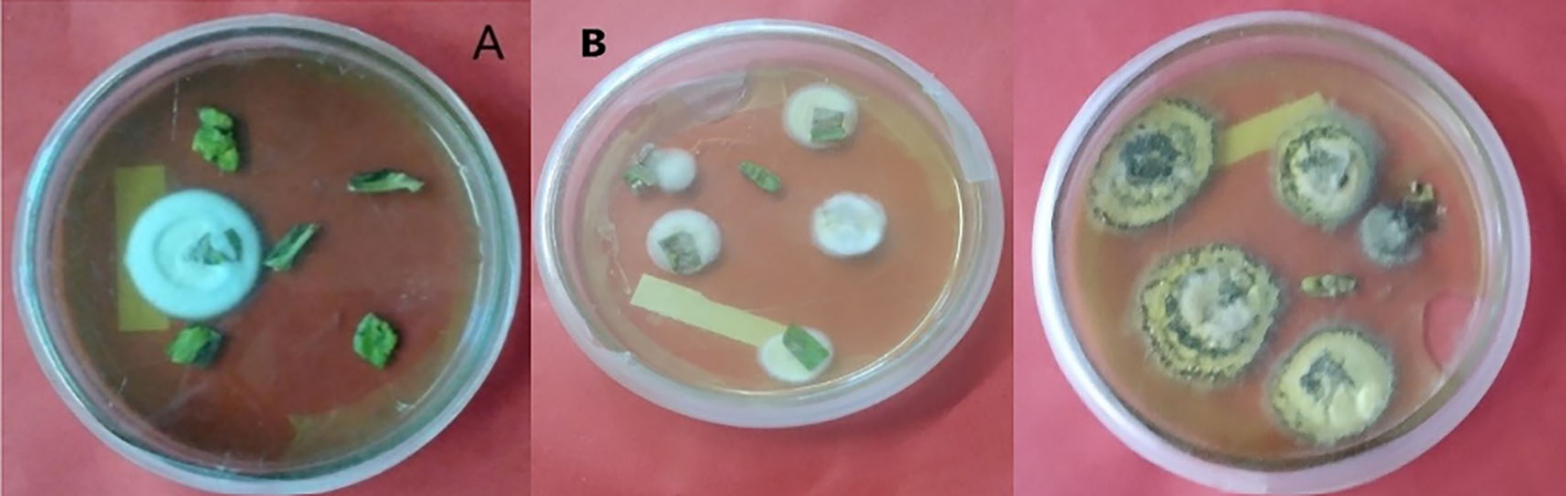
Recovery of isolates from tomato plants 7-DPI by leaf spray techniques. A = colony color (front) of *B. bassiana*, B = colony color (front) *M. robertsii.*

In the case of *M. robertsii,* successful endophytic colonization of tomato plants was obtained by the leaf spray method with 53.33% at 7-DPI in leaves ( Figure 3), and no fungal colonization occurred in root tissues after 28 days of inoculation. This suggested that the recovery of *B. bassiana* and *M. robertsii* from inoculated hosts decreased from the site of inoculation in plant tissue over time.

***Seedling inoculation***

In contrast to the leaf spray technique, this study showed tomato plants were successfully colonized by *B. bassiana* isolate APPRC-27 28 days post inoculated, with 65% of colonization in roots by the seedling inoculation technique ( Table 4). The highest endophytic percent of colonization was detected from tomato roots, followed by stems, in this method after 28 days of inoculated by all isolates.

**Table 4.** Mean (±SE) percentage of endophytic colonization of entomopathogenic fungi in tomato plants.

Isolate	Day	Seedling inoculation			Root dipping		
		Leaves	Stems	Roots	Leaves	Stems	Roots
*M. robertsii* K102	7	8.33±0.22 ^c^	11.10±0.93 ^c^	18.33±0.80 ^c^	9.99±0.66 ^c^	21.67±1.00 ^b^	33.33±0.31 ^b^
	14	13.33±0.31 ^c^	18.33±0.80 ^b^	28.33±0.95 ^b^	7.50±0.41 ^b^	18.33±0.80 ^b^	26.00±1.16 ^b^
	21	20.00±0.75 ^b^	31.67±0.88 ^b^	36.67±0.83 ^c^	3.33±0.33 ^b^	10.00±0.18 ^b^	13.33±0.50 ^b^
	28	28.33±0.91 ^c^	38.33±0.73 ^c^	45.00±0.80 ^c^	0.00±0.00 ^b^	5.00±0.19 ^b^	8.33±0.19 ^b^
*M. robertsii* K61	7	11.67±0.29 ^b^	17.23±0.72 ^b^	26.67±1.13 ^b^	23.33±0.85 ^a^	38.33±0.84 ^a^	43.33±0.54 ^a^
	14	25.00±0.37 ^a^	30.00±0.77 ^a^	45.00±0.92 ^a^	16.16±0.27 ^a^	23.33±0.85 ^a^	32.33±0.44 ^a^
	21	23.33±0.85 ^b^	28.00±0.78 ^b^	50.00±0.76 ^b^	7.43±0.18 ^a^	13.33±0.41 ^a^	21.67±0.47 ^a^
	28	33.33±0.77 ^b^	50.00±0.79 ^b^	71.67±0.98 ^a^	3.33±0.11 ^a^	8.33±0.15 ^a^	10.00±0.40 ^a^
*B. bassiana APPRC27*	7	15.00±0.45 ^a^	21.67±0.25 ^a^	35.00±0.74 ^a^	10.13±0.08 ^b^	13.33±0.49 ^c^	15.00±0.58 ^c^
	14	21.67±0.31 ^b^	33.33±0.30 ^a^	40.00±1.31 ^a^	5.00±0.42 ^c^	8.33±0.31 ^c^	13.33±0.50 ^c^
	21	35.00±0.26 ^a^	46.67±0.86 ^a^	58.33±0.41 ^a^	1.67±0.40 ^c^	3.33±0.57 ^c^	6.67±0.49 ^c^
	28	47.13±0.64 ^a^	55.86±0.52 ^a^	65.00±0.33 ^b^	0.00±0.00 ^b^	0.00±0.00 ^c^	3.33±0.41 ^c^
*Control*	7	0.00±0.00 ^d^	0.00±0.00 ^d^	0.00±0.00 ^d^	0.00±0.00 ^d^	0.00±0.00 ^d^	0.00±0.00 ^d^
	14	0.00±0.00 ^c^	0.00±0.00 ^c^	0.00±0.00 ^c^	0.00±0.00 ^d^	0.00±0.00 ^d^	0.00±0.00 ^d^
	21	0.00±0.00 ^c^	0.00±0.00 ^c^	0.00±0.00 ^d^	0.00±0.00 ^d^	0.00±0.00 ^d^	0.00±0.00 ^d^
	28	0.00±0.00 ^d^	0.00±0.00 ^d^	0.00±0.00 ^d^	0.00±0.00 ^b^	0.00±0.00 ^c^	0.00±0.00 ^d^

**Figure 4.**
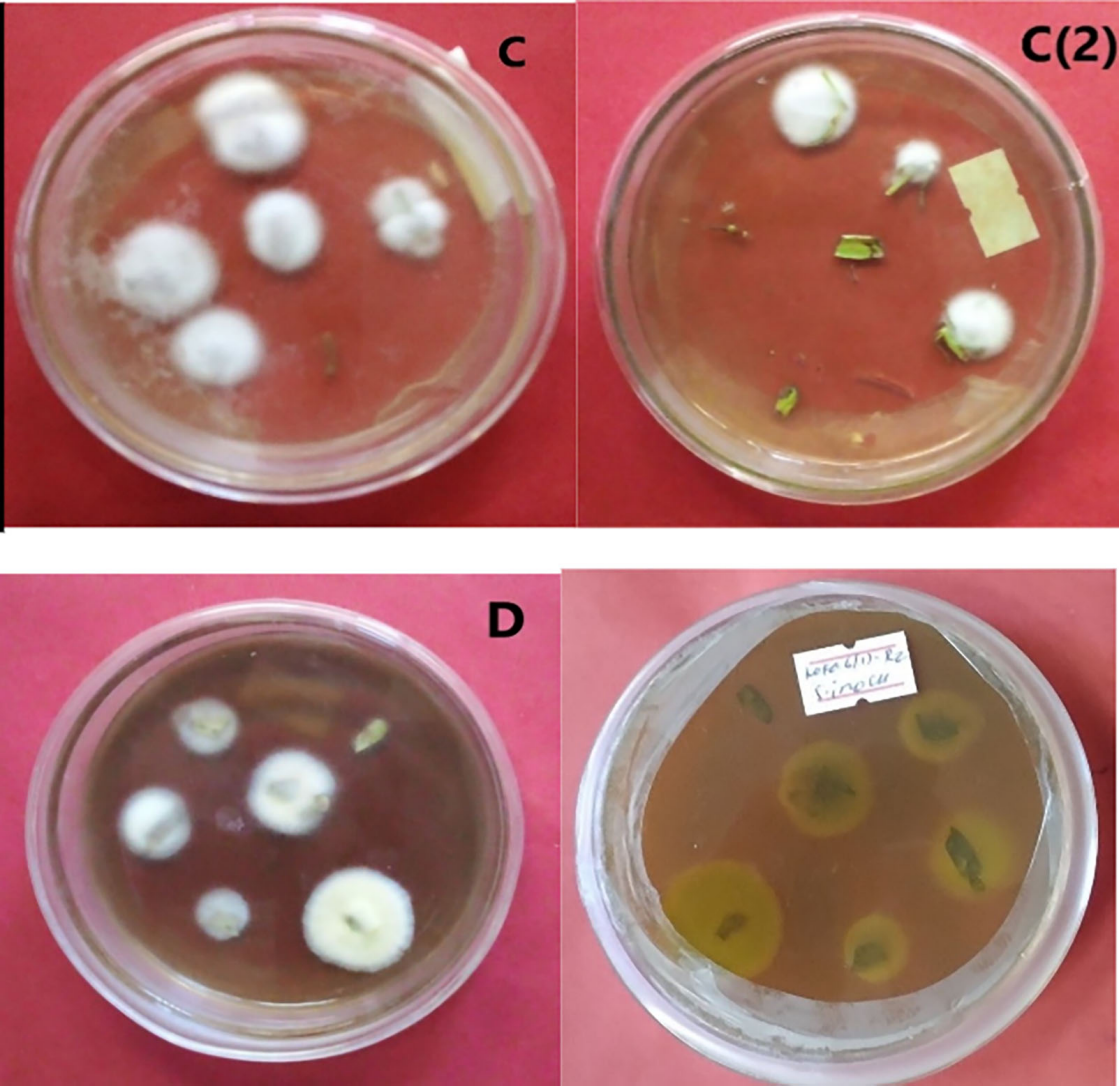
Morphology of *B. bassiana* and *M. robertsii* after recovery from tomato. C = colony color of isolate APPRC-27 from tomato roots; C (2) colony color of isolate APPRC-27 from tomato leaves; D = colony color (front-to-left, back-to-right) of isolate K-61.

Maximum colonization mean percent (71.67% SE±0.98) was obtained from *M. robertsii* isolate K-61 at 28 DPI from tomato roots, whereas minimum colonization was obtained from *M. robertsii* isolate K-102 (8.33%±SE ±0.22) in leaf tissue at 7 DPI ( Figure 4).

This result estimated that the persistence of *B. bassiana* and *M. robertsii* in the tomato plants increased up to 28 days after inoculation by the seed inoculation technique.

In all techniques of inoculation, there was a variation in endophytic colonization of tomato plants by *M. robertsii* and *B. bassiana* over time, and there was no fungal detection from uninoculated tomato plants. From the results, we can suggest that inoculation techniques greatly affect the movement and persistence of fungal isolates through the plant tissues of tomatoes. There is a significant difference in the interaction of inoculation techniques for the establishment of fungal isolates as endophytes within plant tissues. Accordingly, the leaf spray inoculation technique was a successful method for the endophytic colonization of entomopathogenic fungi in tomato plants, which were used against tomato leaf miner ( *T. absoluta*). This study suggested that the establishment of *M. robertsii* and one *B. bassiana* as endophytes in the tomato plant reduces the population of *T. absoluta by* interfering with the feeding ability of larvae.

***Root dipping technique***

In the case of the root dipping technique of inoculation, the highest endophytic colonization of tomato plants was recorded by *M. robertsii* isolate K-61 after 7 days (43.33%) in root tissues, and it declined to 10% at 28 DPI. The Lowest endophytic colonization (3.33%) was registered in tomato plants treated with *B. bassiana* isolate APPRC-27 after 28 days of inoculation in root tissues, and no fungal colonization was detected in stems and leaves on the same day ( Table 4).

The result of interaction between two variables (inoculation techniques and days DPI at 7, and 28) showed that there is a highly significant difference in endophytic colonization of tomato leaves by fungal isolates (DF = 3, F = 1001.80, P < 0.0001). As a result, the leaf spray technique shows an increase in endophytic colonization by entomopathogenic fungi up to 7 DPI (38.30%) and a decline to 14.86% at 28 days in the leaves of the tomato plant. However, Seedling inoculation technique increased over time and higher endophytic colonization of entomopathogenic fungi obtained at 28 DPI (27.61%) ( Figure 4).

In the case of root colonization of tomato plants by entomopathogenic fungi, the interactions of two variables show that the seedling inoculation technique shows a maximum endophytic colonization at 28 DPI.

### Discussions

In this study, selected isolates were screened for their pathogenicity against *T. absoluta* larvae. Pathogenicity tests revealed that all isolates showed 72.5% up to 97.5% mean mortality of *T. absoluta* larvae at a concentration of 1 × 10 ^8^ conidial/ml after 7 days of inoculation. This result agrees with the study conducted by Ayele *et al.* (2020), where maximum mortality rates (95%) were registered on the 2 ^nd^ larval instar of *T. absoluta* by *M. robertsii* isolate AAUM39 at a concentration of 1 × 10 ^7^ conidial/ml 7 days post-inoculation. Shibiru and Getu (2017) also reported that the mortalities caused by *B. bassiana* isolate at the different concentrations ranged from 79.17% to 95.83% under laboratory conditions, and 87.50% mortality was registered by *M. robertsii* after 7 days of treatment application at 2.5 × 10 ^9^ conidia/ml.

The entomopathogenic *B. bassiana* fungus could cause larval mortality of up to 95% compared to chemical treatment of 88% ( El Kichaoui *et al*., 2016). In this study, certain selected isolates were evaluated for their virulence against *T. absoluta* larvae by direct contact. *B. bassiana* caused the highest cumulative mortality of larvae (100%) at 10 DPI at a concentration of 1x10 ^8^ conidia/ml. A similar study indicated that direct contact by leaf spraying showed a higher mortality rate and a lower MST value than indirect contact of *T. absoluta* larvae with conidia ( Allegrucci *et al*., 2017).

By introducing entomopathogenic fungi as endophytes, the pathogen could be shielded from unfavorable environmental factors, lessening the impact of UV radiation and the developmental constraints of low humidity ( Resquín-Romero *et al*., 2016). Vega *et al*. (2008b) reported that 90 genera of entomopathogenic fungi are known, and most of them exist as endophytes in plants, but only 12 species have been tested as biocontrol agents.

In the present work, B. *bassiana* and *M. robertsii* were successfully colonized tomato plants endophytically by using different artificial inoculation techniques. The highest endophytic colonization was attained by B. *bassiana* isolate APPRC-27 (76.67%) through leaf spray technique, followed by *M. robertsii* isolate K-61 (71.67%) by seedling inoculation technique at 7 and 28 DPI, respectively. This revealed that the colonization of *B. bassiana* observed in tomato leaves at 7-DPI was 29.44% by the leaf spray method, which was reported as the most effective method of inoculation for the establishment of entomopathogenic fungi as an endophyte ( Allegrucci *et al*., 2017). This may be due to the stomata structures, which are the main entry and channel for many pathogenic fungi ( Shikano *et al*., 2017; Gonzalez *et al*., 2021). However, *B. bassina* may have the potential to invade plant tissues if stomata are encountered under unfavorable conditions ( Wagner & Lewis, 2000).

This study revealed that endophytic colonization of fungal conidia in tomato plants was increased over time up to 30 DPI by using the seedling inoculation technique, and the highest endophytic colonization was detected from tomato root tissues after 28 days of inoculation. The result is consistent with the root tissues and stems displayed the highest percent of colonization at 7 DPI, with 80%, and at 30 DPI, all samples from all parts of the tomato plants were 100% positive for fungal colonization of B. *bassiana* isolate LPP139 by the seedling inoculation method, which results in relatively rapid and lasting colonization of tomato plants ( Silva *et al*., 2020).

However, endophytic colonization of tomato plants by fungal conidia decreased over time by using leaf spray and root dipping techniques of inoculation for both species of tested isolates. In the case of root dipping, the highest recovery of endophytic fungi was obtained by *M. robertsii isolate* K-61 (43.33%) after 7 days of inoculation. Similarly, successful endophytic colonization of tomato plants was achieved through the root dipping method with 12.22% mean values in root tissues at 7 DPI by *B. bassiana* ( Allegrucci *et al*., 2017).

The result is consistent with the fact that *Metarhizhium* species prefer root tissues to leaves as endophytic colonization after artificial inoculation, while *B. bassiana* prefers aerial plant parts ( Behie *et al*., 2015). *M. robertsii* has a well-established relationship with the rhizosphere ( Leger, 2008; Bruck, 2005). However, successful endophytic colonization of *B. bassiana* occurred in tomato root tissue at 37.75% at 7 dpi by the root dipping method ( Mwamburi, 2021).

In all inoculation techniques, this study revealed that *B. bassiana* and *M. robertsii* can migrate endophytically from the inoculated sites and colonize other plant tissues in the tomato plant. This supports the systematic translocation and colonization of fungal inoculums in plant tissues from sites inoculated to other plant tissues ( Tefera and Vidal, 2009; Sánchez-Rodríguez *et al.,* 2015; Omukoko, 2018; Sinno *et al*., 2021). Elena *et al.* (2011) also reported that *M. robertsii* was capable of moving within the plant and B. *bassiana* was able to colonize corn and maize within plant tissues by stem injection technique.

## Conclusions

*T. absoluta* is a major pest of tomato plants and difficult to control due to its mine-feeding nature in mesophyll tissues. As a result, using entomopathogenic fungi such as *B. bassiana* and *M. robertsii* as endophytes is a novel and eco-friendly method for the management of tomato leaf miner due to its migratory potential, deployment, and long persistence over chemical insecticides.

Isolates with high germination percent and maximum spore production were highly virulent to 2nd-instar larvae of *T. absoluta.* Accordingly, *B. bassiana* isolate APPRC-27 was highly virulent to *T. absoluta* by direct contact method with 100% cumulative mortality, followed by *M. robertsii* isolate K-61 (96.49%) at 10-days post-inoculated at a concentration of 1 × 10 ^8^ conidial/ml. Among the tested isolates, the highest colonization percent was achieved by *B. bassiana* isolate APPRC-27 (76.67%) from tomato leaves at 7 days post-inoculation through the leaf spray technique.

This study confirmed that the virulent isolates had the potential to colonize and migrate in tomato plant tissues. However, endophytic colonization reduced over time at 28 days post-inoculation for all isolates by leaf spray and root dipping techniques while increasing by seedling inoculation techniques. The inoculation techniques greatly affect the persistence and establishment of *B. bassiana* and *M. robertsii* as endophytes in tomato plant tissues over time.

Using two *M. robertsii* isolates, K-61 and K-102, and a *B. bassiana* isolate, APPRC-27, is the best biological option against larvae of *T. absoluta.* The leaf spray and seedling inoculation techniques are the most effective with *B. bassiana* isolate APPRC-27 and *M. robertsii* isolate K-61, respectively, for the establishment of fungal conidia as endophytes in the tomato plant tissues against *T. absoluta.*

However, further studies must be conducted on the persistence and mechanism of migration of *B. bassiana* and *M. robertsii* in tomato plant tissues concerning physiological activities within the tomato plant. Moreover, further studies are needed on the factors attributed to the efficacy and endophytic association of *B. bassiana* and *M. robertsii* in tomato and other Solanaceae plants against *T. absoluta* under field conditions. The compatibility of virulent isolates with other management options also needs to be taken into consideration while developing integrated pest management strategies for the control of *T. absoluta.*

## Data Availability

Figshare: Arcsine transformed data, evaluation of inoculation techniques of endophytic entomopathogenic fungi, lt50% https://doi.org/10.6084/m9.figshare.25585323.v1 ( Geremew, 2024). This project contains the following underlying data:
• Arcsine transformed data • evaluation of inoculation techniques of endophytic entomopathogenic fungi • LT50% Data are available under the terms of https://creativecommons.org/licenses/by/4.0/ (CC BY 4.0 DEED).
