# Supplementary material for: Evaluation of endophytic colonization and establishment of entomopathogenic fungi against Tuta absoluta (Lepidoptera: Gelechiidae) in tomato plants
Source: F1000Res. 2024 Jul 16;13:800. [Version 1] doi: 10.12688/f1000research.148658.1 (PMC11347916; doi:10.12688/f1000research.148658.1)
Supplement: Peer review history

# Reviewer report: Reviewer response for version 1

**DOI:** 10.5256/f1000research.162998.r304612

**Published:** 2024-08-26

**Authors:** Preety Tomar

**Affiliations:** 1 Department of Zoology, Akal College of Basic Sciences, Eternal University, Baru Sahib, Himachal Pradesh, India

The research article entitled "Evaluation of endophytic colonization and establishment of entomopathogenic fungi against *Tuta absoluta *(Lepidoptera: Gelechiidae) in tomato plants" is a very good work carried by Greremew et al. The authors have selected an important area of investigation which is the management of leaf miner larvae using entomopathogens. The results are supplemented by tables and figures including nice photographs. The only thing that i want to suggest is about the writing of scientific names. They should be italic and should be written as whole only once after that they should be written as short form. There are some typographical errors though they do not belittle the importance and genuineness of the work.

Is the work clearly and accurately presented and does it cite the current literature?

Yes

If applicable, is the statistical analysis and its interpretation appropriate?

Yes

Are all the source data underlying the results available to ensure full reproducibility?

Yes

Is the study design appropriate and is the work technically sound?

Yes

Are the conclusions drawn adequately supported by the results?

Yes

Are sufficient details of methods and analysis provided to allow replication by others?

Yes

Reviewer Expertise:

Entomopathogens, Biocontrol, Microbes, Crop protection

I confirm that I have read this submission and believe that I have an appropriate level of expertise to confirm that it is of an acceptable scientific standard.

# Reviewer report: Reviewer response for version 1

**DOI:** 10.5256/f1000research.162998.r304615

**Published:** 2024-08-05

**Authors:** Eustachio Tarasco

**Affiliations:** 1 Department of Soil, Plant and Food Sciences, University of Bari “Aldo Moro”, Bari, Italy

Bibliography needs to be increased as well as systematics (each species when reported for the first time in the text should be written in full with Authority and systematics) and details on the origin and characterization of the entomopathogenic fungi used in the experiments. The methodology can be better explained with a more detailed experimental design. Moreover too short conclusions. The last 2 chapters could be better arranged

Is the work clearly and accurately presented and does it cite the current literature?

Partly

If applicable, is the statistical analysis and its interpretation appropriate?

Yes

Are all the source data underlying the results available to ensure full reproducibility?

Yes

Is the study design appropriate and is the work technically sound?

Partly

Are the conclusions drawn adequately supported by the results?

Yes

Are sufficient details of methods and analysis provided to allow replication by others?

Yes

Reviewer Expertise:

microbiological control, insect pathology

I confirm that I have read this submission and believe that I have an appropriate level of expertise to confirm that it is of an acceptable scientific standard, however I have significant reservations, as outlined above.

# Reviewer report: Reviewer response for version 1

**DOI:** 10.5256/f1000research.162998.r304611

**Published:** 2024-08-01

**Authors:** Julner Pachoute

**Affiliations:** 1 Federal University of Tocantins, Palmas, State of Tocantins, Brazil

In the part *"EPF from adverse conditions, the incorporation of EPF in plants as endophytes would appear to be a highly interesting and promising approach (Vega, 2018)"*

**This sentence is not clear, when first mention EPF, it would be better to write it in extension.**

 In the part *: Knowing the endophytic association and establishment of EPF in tomato plants helps to modify the inoculation techniques for plant parts based on the behavior of targeted pests, particularly T. absoluta, which lives in the mesophyll tissue of the tomato plant.*

**Can yo add some researches about EPF on tomato, especially about B. bassiana and M. robertsi?**

**Did you do a literature review about the biological control of T. absoluta?**

**Can you add in the text?**

 In the part: *10 km to the west, at a geographical coordinate of 8°59’ N latitude and 37.85°E longitude, with an altitude of 2100 meters above sea l*

**Revise the coordinates?**

 In the part: *Conidia were obtained from pure cultures grown on the 65g of Sabouraud Dextrose Agar (SDA) Microxpress® REF: 2*

**Conidia of what? Please cite the fungus.**

 In the part: *The mass rearing of T. absoluta was established in 2021 from larvae collected from tomato plants without any history of pesticides in fields surrounding Ambo areas. The Roman VF variety tomato plant was grown in the glasshouse to maintain the life cycle of T*

**Please describe the collet of the larvae T. absoluta**

 In the part: *High-virulent B. bassiana and M. robertsii isolates that were prescree*

**Can you talk more about the origin of the isolates**

 In the section: *Currently, EPF is more promising than chemical pesticides for the control of pests in Ethiopia due to pesticide resistance.*

**Are you sure? Do you have more augment ou reference to support this thesis.**

 In the section: *The result is consistent with the fact that Metarhizium species prefer root tissues to leaves as endophytic colonization after artificial inoculation, while B. bassiana prefers aerial plant parts (Behie et al., 2015). M. robertsii has a well-established relationship with the rhizosphere (Leger, 2008; Bruck, 2005). However, successful endophytic colonization of B. bassiana occurred in tomato root tissue at 37.75% at 7 dpi by the root dipping method (Mwamburi, 2021).*

**Can you explain this preference?**

 In the section: *all inoculation techniques, this study revealed that B. bassiana and M. robertsii can migrate endophytically from the inoculated sites and colonize other plant tissues in the tomato plant. This supports the systematic translocation and colonization of fungal inoculum's' in plant tissues from sites inoculated to other plant tissues (Tefera and Vidal, 2009; Sánchez-Rodríguez et al., 2015; Omukoko, 2018; Sinno et al., 2021). Elena et al.(2011) also reported that M. robertsii was capable of moving within the plant and B. bassiana was able to colonize corn and maize within plant tissues by stem injection technique.*

**Is it a good idea to discuss the results comparing different species?**

 In the section: *the potential to colonize and migrate in tomato plant tissues. However, endophytic colonization reduced over time at 28 days post-inoculation for all isolates by leaf spray and root dipping techniques while increasing by seedling inoculation techniques.*

**Can you explain why?**

**Provide photos with scale bar.**

Is the work clearly and accurately presented and does it cite the current literature?

Partly

If applicable, is the statistical analysis and its interpretation appropriate?

Yes

Are all the source data underlying the results available to ensure full reproducibility?

Yes

Is the study design appropriate and is the work technically sound?

Yes

Are the conclusions drawn adequately supported by the results?

Yes

Are sufficient details of methods and analysis provided to allow replication by others?

Yes

Reviewer Expertise:

I have a Ph. D in plant production. I did my thesis on biological control, entomopathogenic fungi, endophytic colonization

I confirm that I have read this submission and believe that I have an appropriate level of expertise to confirm that it is of an acceptable scientific standard, however I have significant reservations, as outlined above.

## References

[1] : Plant tissue localization of the endophytic insect pathogenic fungi Metarhizium and Beauveria. *Fungal Ecology* .2015;13: 10.1016/j.funeco.2014.08.001 112-119 10.1016/j.funeco.2014.08.001

[2] : Metarhizium seed treatment mediates fungal dispersal via roots and induces infections in insects. *Fungal Ecology* .2014;11: 10.1016/j.funeco.2014.05.005 122-131 10.1016/j.funeco.2014.05.005

[3] : Endophytic fungi, Beauveria bassiana and Metarhizium anisopliae, confer control of the fall armyworm, Spodoptera frugiperda (J. E. Smith) (Lepidoptera: Noctuidae), in two tomato varieties. *Egyptian Journal of Biological Pest Control* .2021;31(1) : 10.1186/s41938-020-00357-3 10.1186/s41938-020-00357-3

[4] : Effect of inoculation method and plant growth medium on endophytic colonization of sorghum by the entomopathogenic fungus Beauveria bassiana. *BioControl* .2009;54(5) : 10.1007/s10526-009-9216-y 663-669 10.1007/s10526-009-9216-y

[5] : Evolution and emergence of plant viruses. *Adv Virus Res* .2014;88: 10.1016/B978-0-12-800098-4.00003-9 161-91 10.1016/B978-0-12-800098-4.00003-9 24373312

# Reviewer report: Reviewer response for version 1

**DOI:** 10.5256/f1000research.162998.r304609

**Published:** 2024-07-29

**Authors:** Yordanys Ramos

**Affiliations:** 1 Instituto de Investigaciones Agropecuarias y Forestales, Universidad Michoacana de San Nicolas de Hidalgo, Morelia, Michoacán, Mexico

The submitted manuscript describes how the entomopathogenic fungi *Beauveria bassiana* and *Metarhizium robertsii *are capable of establishing themselves as endophytes in tomato plants through different artificial inoculation methods while also causing virulence to *Tuta absoluta* larvae. The results contribute to the integrated management of this pest worldwide. However, there are some aspects that need improvement.

**Title**

 The authors should consider restructuring the title to make it more informative. An example:

 Endophytic colonization and establishment of *Beauveria bassiana *and *Metarhizium robertsii *against *Tuta absoluta *(Lepidoptera: Gelechiidae) in tomato plants.

**Abstract**

 In the results section of the abstract, it is preferable to refer to the isolates as *B. bassiana* APPRC-27 and M. robertsii K-61.

*B. bassiana* and *M. robertsii* should be italicized. Correct this throughout the entire document. Additionally, this should be applied to all Latin names used.

**Keywords**

 In the keywords, avoid repeating words that were previously used in the title of the article, such as colonization, entomopathogenic fungi, and *Tuta absoluta*.

**Introduction**

 The statement in the introduction that says, "Currently, EPF is more promising than chemical pesticides for the control of pests in Ethiopia due to pesticide resistance," needs a citation to support it.

**Methods**

 Conidia were obtained and incubated for 14 days at 27 ± 1°C under dark conditions. At what relative humidity percentage were they conditioned?

 Were conidia germination tests conducted? Was there any criterion for conidia viability to use them in the bioassays?

 The authors mention that: "Tomato leaves surface disinfection was done by washing with 1% sodium hypochlorite for 2 min, 2 min in ethanol (70%), and rinsing 3 times with sterile distilled water." However, how did they verify that the sterilization was successful? Typically, this is done by inoculating part of the content of the final rinse water onto PDA or SDA culture media.

 Please specify the dimensions of the plastic plates used to place the tomato leaves inoculated with the entomopathogenic fungi.

 You said: "A control treatment was sprayed with Tween 80 (0.001%) and incubated under the same conditions." It should be "treatment was sprayed with sterile distilled water + Tween 80 (0.001%)."

 The authors mention that dead larvae were surface sterilized, but it does not specify how. Was it the same sterilization method as for the tomato leaves mentioned earlier, or was a different method used? Please clarify to avoid ambiguities.

 The text should provide more information: "Plates with larval cadavers were moistened daily with distilled sterile water to enhance mycosis development over the cadavers and incubated at room temperature”. Were they incubated in total darkness?

 Please add the magnification of the stereoscope used.

 Specify the type of antibiotic and its concentration used in the culture medium when planting the leaves to detect the success of the endophytic establishment of the entomopathogenic fungi.

**Results**

 The authors mention that "There is a highly significant difference among treatments in mycosis percentage on dead cadavers." Statistical data confirming this statement should be provided. This should be included whenever a comparison is being made.

 In Figure 2, the standard errors should be included in the lines for each treatment.

**Conclusions**

 In the conclusions, the authors should avoid repeating the results obtained and be more concise.

Is the work clearly and accurately presented and does it cite the current literature?

Yes

If applicable, is the statistical analysis and its interpretation appropriate?

Yes

Are all the source data underlying the results available to ensure full reproducibility?

Yes

Is the study design appropriate and is the work technically sound?

Yes

Are the conclusions drawn adequately supported by the results?

Partly

Are sufficient details of methods and analysis provided to allow replication by others?

Partly

Reviewer Expertise:

Biological Control, Entomology, Microbiology, Integrated Pest Management, Molecular Biology

I confirm that I have read this submission and believe that I have an appropriate level of expertise to confirm that it is of an acceptable scientific standard, however I have significant reservations, as outlined above.
